# Luteolin Induces Carcinoma Cell Apoptosis through Binding Hsp90 to Suppress Constitutive Activation of STAT3

**DOI:** 10.1371/journal.pone.0049194

**Published:** 2012-11-08

**Authors:** Jin Fu, Dan Chen, Bo Zhao, Zhihui Zhao, Jiahong Zhou, Yimiao Xu, Yinqiang Xin, Chang Liu, Lan Luo, Zhimin Yin

**Affiliations:** 1 Jiangsu Province Key Laboratory for Molecular and Medicine Biotechnology, College of Life Science, Nanjing Normal University, Nanjing, People’s Republic of China; 2 State Key Laboratory of Pharmaceutical Biotechnology, School of Life Sciences, Nanjing University, Nanjing, People’s Republic of China; 3 College of Chemistry and Material Science, Nanjing Normal University, Nanjing, People’s Republic of China; 4 Center for Analysis and Test, Nanjing Normal University, Nanjing, People’s Republic of China; Technische Universitaet Muenchen, Germany

## Abstract

**Background:**

Abnormal activity of STAT3 is associated with a number of human malignancies. Hsp90 plays a central role in stabilizing newly synthesized proteins and participates in maintaining the functional competency of a number of signaling transducers involved in cell growth, survival and oncogenesis, such as STAT3. Hsp90 interacts with STAT3 and stabilizes Tyr-phosphorylated STAT3. It has been reported that luteolin possesses anticancer activity through degradation of Tyr^705^-phosphorylated STAT3.

**Methodology/Principal Findings:**

We found that overexpression of Hsp90 inhibited luteolin-induced degradation of Tyr^705^-phosphorylated STAT3 and luteolin also reduced the levels of some other Hsp90 interacting proteins. Results from co-immunoprecipitation and immunoblot analysis demonstrated that luteolin prevented the association between Hsp90 and STAT3 and induced both Tyr^705^- and Ser^727^-phosphorylated STAT3 degradation through proteasome-dependent pathway**.** The molecular modeling analysis with CHARMm–Discovery Studio 2.1(DS 2.1) indicated that luteolin could bind to the ATP-binding pocket of Hsp90. SPR technology-based binding assay confirmed the association between luteolin and Hsp90. ATP-sepharose binding assay displayed that luteolin inhibited Hsp90-ATP binding.

**Conclusions/Significance:**

Luteolin promoted the degradation of Tyr^705^- and Ser^727^-phosphorylated STAT3 through interacting with Hsp90 and induced apoptosis of cancer cells. This study indicated that luteolin may act as a potent HSP90 inhibitor in antitumor strategies.

## Introduction

Flavonoids are polyphenolic compounds occurring in a wide range of plants, which can efficiently suppress the proliferation of tumor cells and induce apoptosis by blocking cell cycle progression [1], [2], [3], [4], [5]. Luteolin, 3′,4′,5,7-tetra-hydroxyflavone, is the flavone subclass of flavonoids isolated from celery, perilla leaf, camomile tea and green pepper [6]. Recently, luteolin has been found to possess a potent anticancer activity in several experiments, and even at low dosage it displays a marked effect on killing malignant cells [7], [8].

It has been reported that luteolin could induce degradation of Tyr^705^-phosphorylated STAT3 (Signal transducer and activators of transcription 3) [9]. STAT3 can be activated through tyrosin and/or serine phosphorylation by diverse stimulations, and activated STAT3 enters into nucleus and works coordinately with other transcriptional co-activators or transcription factors to initiate transcription [10], [11]. Constitutive activation of STAT3 is a requirement for the oncogenic transforming property [12]. In fact, the antiapoptotic genes encoding c-Myc, Bcl-2, Bcl-xl, cyclin D1, and survivin are downstream targets of STAT3 [13]. Abnormal activity of STAT3 is associated a number of human malignancies, including hematologic, breast, head, neck, and prostate cancers.

Heat shock protein (Hsp) 90, an ATP-dependent protein, may function as a stabilizer of Tyr-phosphorylated STAT3 by directly interacting with it [14]. Hsp90, interacting with a variety of cytoplasm proteins including transcription factors, hormone receptors and proteins kinases [15], [16], [17], is one of the most abundant and ubiquitous molecular chaperones, and has been shown to make nascent client proteins fold correctly, sustain the stability and function of client proteins. Inhibition of Hsp90 activity will lead to degradation of its client proteins in an ubiquitin-proteasome-dependent pathway and disruption of their function [18], [19], and consequently prevent tumor growth. In fact, many client proteins of Hsp90 are crucial in oncogenesis, such as Her-2, Akt, STAT3, and p53 [20], [21]. Hsp90 is increasingly recognized as an important target for molecular cancer therapy due to its role in regulating key proteins in cell growth, survival, and differentiation pathways. Frequent overexpression of Hsp90 in solid and hematologic tumors also suggests the importance of this chaperone in oncogenesis [22].

In the past few years, the various Hsp90-specific inhibitors has been reported, which include benzoquinone ansamycins, such as geldanamycin (GA) derivatives, radicicol (RAD) derivatives, purine scaffold inhibitors, dihydroxyphenylpyrazoles, and small peptides [23], [24], [25]. The natural compounds GA and RAD were described as specific inhibitors of Hsp90 by tightly binding the ATP-binding pocket of Hsp90, which leads to destabilization of Hsp90 complexes with its interacting proteins, rendering them available for proteosomal degradation [26]. GA posses potent and broad anti-cancer properties in vivo, it is not used clinically because of the serious liver and kidney toxicity [21], [27]. Some derivatives of GA, such as 17-Allylamino-17-demthoxygeldanamycin (17-AAG) are now in clinical trials for cancer. Although certainly effective in many tumor models, in clinic 17-AAG is faced with several limitations, which include solubility, stability, and hepatotoxicity [28]. Thus the toxicities of Hsp90 inhibitors must be considered in anticancer therapeutic strategy.

Thus, it is not surprising that new Hsp90 inhibitors are under development for cancer therapy. Several flavonoids have been investigated for their activities to interact with Hsp90 [ ], but the central role of Hsp90 in luteolin anticancer effects remains unclear.Here we revealed a novel mechanism by which luteolin promoted apoptosis of HeLa and MCF-7 cells. Our investigation demonstrated that luteolin could bound to ATP pocket of Hsp90 to block the association between ATP and Hsp90. Therefore luteolin make STAT3 dissociate from Hsp9,and consequently, induced degradation of Tyr^705^-phosphorylated STAT3 and Ser^727^- phosphorylated STAT3.

## Materials and Methods

### Agents

Polyclonal antibodies against caspase-3, PARP, cleaved-PARP, β-actin, phospho-STAT3 (Tyr705/Ser727), STAT3, phospho-STAT1 (Tyr701), phospho-STAT5 (Tyr649), STAT5, α-tublin, c-Myc, Akt, and H3 were obtained from Cell Signaling Technology. Antibodies against Hsp70 and Hsp90 were from Bioworld Technology, MN. Antibodies against IKKα and IKKβ were from BD. Monoclonal antibodies against HA-tag and Flag-tag were from Roche Applied Science. Secondary antibodies coupled to IRDy800 flurophore for use with the Odyssey Infrared Imaging System were purchased from Rockland. Secondary antibody coupled to Alexa Fluor 488 was from Invitrogen. Alexa Fluor 555 phalloidin and DAPI were from Invirogen. Luteolin, GA, Ethanol, DMSO and MG-132 were from Sigma. CHX, Act D, and z-VAD-fmk were obtained from Calbiochem.

### DNA Constructs

The pcDNA3-HA-Hsp90 plasmid was generous gift from Dr. Chen Wang (Chinese Academy of Sciences, Shanghai, PR China). The pSuper-Hsp90i plasmid was kindly provided by Dr. Kou-Juey Wu (National Yang-Ming University, Taipei, Taiwan). The pcDNA3-Flag-Hsp70 was constructed by PCR from human embryonic liver cDNA library using appropriate restrictive enzymes. The nonsense and antisense Hsp70 oligonucleotides were kindly provided by Dr. Huaqun Chen (Nanjing Normal University). All expression vectors were sequenced to confirm.

### Cell Culture and Transfection

Human cervical cancer cells (HeLa), two human hepatoma cell lines (HepG2, Hep3B), human embryonic kidney (HEK293), human liver cells (WRL-68), and breast cancer cell lines (MCF-7) obtained from American Type Culture Collection (ATCC) were cultured in Dulbecco’s modified Eagle’s medium (Gibco) containing 10% fetal bovine serum (Gibco), 100 U/ml penicillin and 100 µg/ml streptomycin at 37°C with 5% CO2. Human XJH B Lymphocyte purchased from the Institute of Biochemistry and Cell Biology, the Chinese Academy of Sciences (Shanghai, People’s Republic of China), were maintained at 37°C and 5% CO_2_ in complete RPMI 1640 (Gibco) supplemented with 10% fetal bovine serum (Gibco) and antibiotics (100 U/ml penicillin and 100 µg/ml streptomycin). Transient transfection was performed using Lipofectamine™ LTX and Plus™ Reagent (Invitrogen) according to the manufacture’s instructions. In all cases, the total amount of DNA was normalized by the empty control plasmids.

### Cell Cytotoxicity Assays

Cell cytotoxicity assays using CytoTox-Glo™ Cytotoxicity Assays kit (Promega) according to the manufacture’s instructions. Briefly,cells were seeded at 10,000 cells/well in 50 µl culture medium, and after 6 hour incubation at 37°C in 5% CO2,. Cells were incubated in culture medium with indicated concentration of luteolin for 24 h. Each well was added with 50 µl CytoTox-Glo™ Cytotoxicity Assay Reagent. After brief mix, the plate was placed at room temperature for 15 minutes and the dead cells signals were measured. After that, 50 µl Lysate Reagent was added to each well to achieve complete cell lysis. Luminescence was measured after 15 minutes of incubation at room temperature. The luminescent signal was adjusted to reflect the “live cell” contribution by subtracting the initial dead cell signal.

### Immunofluorescence Confocal Laser Scanning Microscopy

HeLa cells, grown on Lab-Tek Chamber Slides (Nalge Nunc Int, Naperville, IL), were fixed with 4% paraformaldehyde for 15 minutes at room temperature, and then washed in PBS containing 0.05% Tween 20 (PBS-T). Nonspecific reactions were blocked with ODYSSEY Blocking Buffer (LI-COR) and then incubated with an anti-cleaved-PARP antibody at 4°C overnight. After washing in TBST, the specimens were immunostained with anti-mouse alexa fluor 488 secondary antibody (Molecular Probes, Invitrogen) for 1 h at room temperature, and then stained with Alexa Fluor 555 phalloidin derivatives for labeling F-actin and DAPI. (Confocal laser scanning microscopeLecia TCS SP2/AOBS) The negative stain was performed by omitting the primary antibody in the above experiment.

### Co-immunoprecipitation and Immunoblot Analysis

Cell lysates were centrifuged (15,000×g) at 4°C for 15 min. Proteins were immunoprecipitated with indicated antibodies, respectively. The precleared Protein A/G Plus-Agarose beads (Santa Cruz Biotechnology) or protein agarose beads (Roche Applied Science) were incubated with immunocomplexes for 2 h and washed four times with lysis buffer. The immunoprecipitates were subjected to SDS-PAGE followed by transferring onto polyvinylidene difluoride (RocheApplied Science) or nitrocellulose membranes (Hybond-C, Amersham Biosciences). The antibody–antigen complexes were visualized by the LI-COR Odyssey Infrared Imaging System according to the manufacturer’s instruction using IRDye800 flurophore-conjugated antibody (LI-COR Biosciences, Lincoln, NE).Quantification was directly performed on the blot using the LI-COR Odyssey Analysis Software. Aliquots of whole cell lysates were subjected to immunoblotting analysis to confirm appropriate level of proteins. The antibody–antigen complexes were visualized by chemiluminescence method using TMB immunoblotting system (Promega, Madison, WI, USA).

### Molecular Modeling

The crystal structure of Hsp90 used for molecular modeling was obtained from Protein Data Bank (PDB). In order to optimize hydrogen positions, we removed interatomic bumps and corrected the covalent geometry to make the structure of Hsp90 to be energy-minimized within the CHARMm force filed. Water and all other HETATM residues were removed from the Hsp90 PDB file. Polar hydrogen atoms and Gasteiger charges were added to prepare the Hsp90 molecule for docking. Protein-ligand docking was carried out with the flexible docking tool in the Discovery Studio 2.1 (DS 2.1) software of Accelrys Company in America. The flexible of the residues which surround the active site of Hsp90 and the ligand were all considered in the docking procedure. In this work, the selection of flexible residues for the induced fit was based on the active site of the Hsp90.

### SPR Technology-based Binding Assay

The SPR experiment was performed according to the manufacturer’s instruction. In brief, Hsp90 was immobilized on a CM 5 chip (Biacore, Piscataway, NJ) by the amine-coupling method. The dextran surface was activated by injection of NHS/EDC mixture (0.1 M NHS and 0.4 M EDC, 1∶1 mixture) during 7 min. All the binding experiments were performed at 25°C at a continuous flow rate with 10 mM HEPES buffered saline (10 mM HEPES, 150 mM NaCl, 1 mM MgCl2, 1 mMMnCl2, and 5 mM n-octyl-β-D-glucoside, pH 7.4).

### ATP-sepharose Binding Assay

Briefly, the whole HeLa cell extras were preincubated with ice incubation buffer (10 mmol/L Tris-HCl, 50 mmol/L KCl, 5 mmol/L MgCl_2_, 2 mmol/L DTT, 20 mmol/L Na_2_MoO_4_, 0.01% NP40) containing luteolin, geldanamycin and celastrol. Following incubation, pre-equilibrated γ-phosphatelinked ATP-Sepharose (Jena Bioscience GmbH) was added and incubated at 37°C for 30 min with frequent agitation. Sepharose was subsequently washed and analyzed by SDS-PAGE.

### Luciferase Reporter Assays

HeLa cells cultured in 48-well plates were transiently transfected with a luciferase reporter gene containing STAT3-responsive elements. 24 h after transfection, cells were treated with various concentrations of luteolin for 24 h. Protein samples were prepared and the luciferase activity was measured with Luciferase Assay System according to the manufacturer’s protocol (Promega), and analysed by the Luminometer TD-20/20 (Turner Co. Ltd., Sunnyvale, CA, USA). Each transfection was performed at least twice with triplicates.

### Statistics

Analysis of variance (one-way ANOVA) was used to compare the results between two groups. Data are presented as means ± S. D. Differences were considered significant for P<0.05. Immunoblotting analysis experiments were repeated 2–3 times with similar trends.

## Results

### Luteolin Reduces the Level of Phosphorylated STAT3 and Inhibits the Transcriptional Activity of STAT3

**Figure 1:**
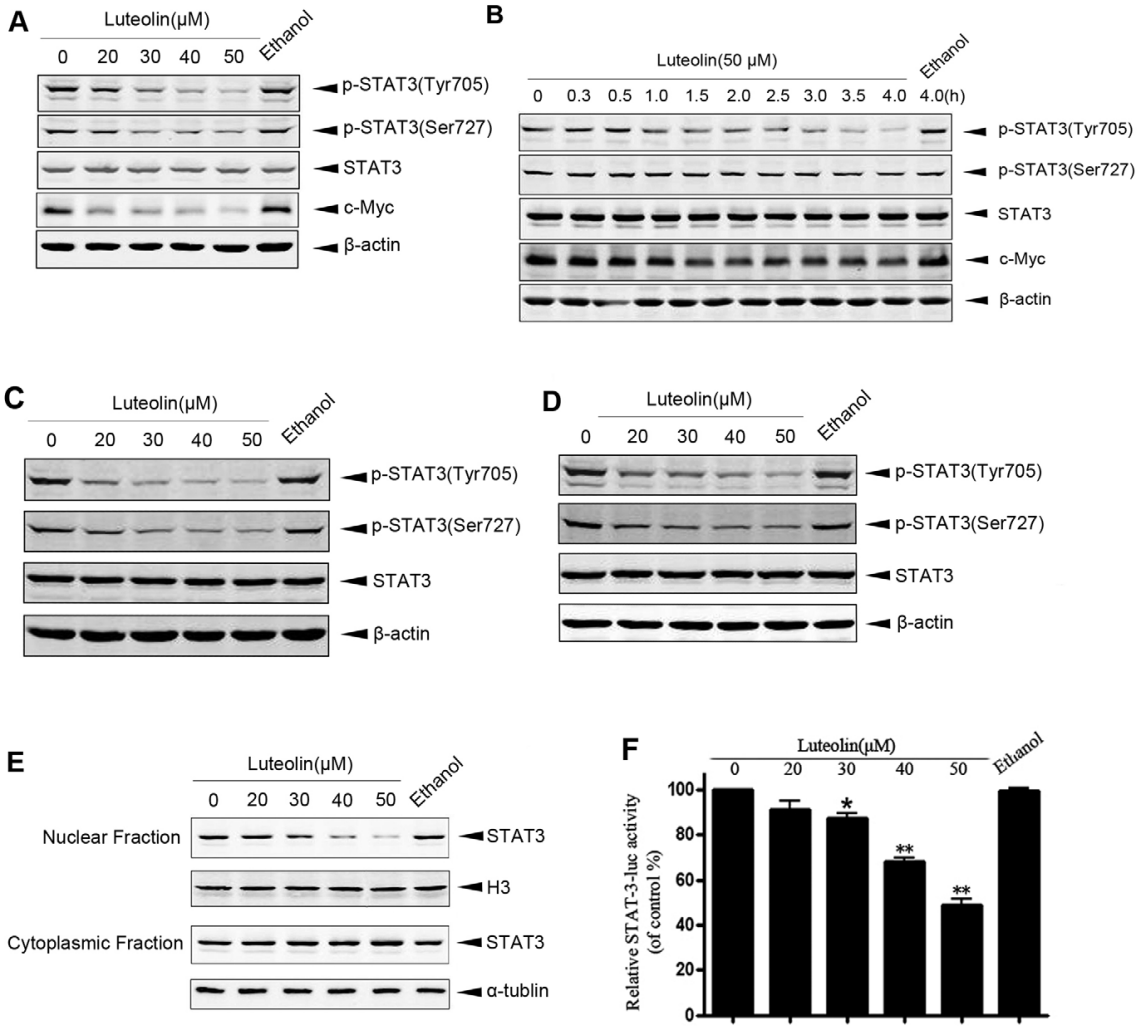
Luteolin inhibits activation of STAT3 in carcinoma cells. A, HeLa cells were treated with indicated concentrations of luteolin for 24 h and then were subjected to Western blot for measuring protein levels of phosphor-STAT3 (tyr705), phosphor-STAT3 (ser727), STAT3 and c-myc respectively. B, HeLa cells were treated with 50 µM luteolin for different time and then were subjected to Western blot by using phosphor-STAT3 (tyr705), phosphor-STAT3 (ser727), STAT3 or c-myc antibody respectively. C, MCF-7 cells were treated with indicated dose of luteolin, and then subjected to Western blot for detecting protein levels of phosphor-STAT3 (tyr705), phosphor-STAT3 (ser727) and STAT3. D, Hep3B cells treated with indicated dose of luteolin and then subjected to Western blot for detecting protein levels of phosphor-STAT3 (tyr705), phosphor-STAT3 (ser727) and STAT3. E, HeLa cells were treated with indicated dose of luteolin respectively for 24 h and then were subjected used for measuring the nuclear and cytoplasmic fraction of STAT3. F, HeLa cells were transfected with STAT3 luciferase reporter plasmids for 24 hours and then treated with luteolin for 24 hours. Luciferase activity was measured by Luminometer TD-20/20. Values were obtained from three independent experiments. _*P<0.05, **P<0.001, compared with the control.

It has been reported that STAT3 participated in the development of a wide variety of human cancer [9], [29], thus we detected phosphorylated STAT3 protein level in HeLa cells under the luteolin treatment condition by Western blot assay. Indeed, luteolin reduced the level of Tyr^705^- and Ser^727^- phosphorylated STAT3 dose-dependently 24 h after being added to cells (Fig. 1A). As shown in Fig. 1A, luteolin induced a notable reduction in the level of Tyr^705^-phosphorylated STAT3, but just induced a mild decrease in Ser^727^-phosphorylated STAT3 level. We then observed when phosphorylated STAT3 began to decrease. Fig. 1B showed that Tyr^705^-phosphorylated STAT3 level reduced 1 h after luteolin treatment and reduced to a very low level after 4 h, but Ser^727^-phosphorylated STAT3 only decreased slightly. The level of total STAT3 showed no changes (Fig. 1A and B). Consistent with this finding, luteolin also induced a decrease of phosphorylated STAT3 in other kinds of malignant cells, including MCF-7 (Fig. 1C) and Hep3B (Fig. 1D) cells.

It has been well documented that cytosolic STATs monomers may be phosphorylated by different stimulus to form tyrosine phosphorylated dimers and then translocate into the nucleus to activate transcription [30]. The results from our experiments demonstrated that treating HeLa cells with luteolin for 24 h led to a dose-dependent decrease of STAT3 nuclear translocation and prolongation of the dwell time of STAT3 in the cytoplasm (Fig. 1E). Our further experiments demonstrated that luteolin weakened STAT3 luciferase reporter activity which also suggested that luteolin inhibited the activity of STAT3 (Fig. 1F). Because oncogenic transcription factor STAT3 up-regulated the tumorigenic genes, such as c-Myc, which contributed to cell cycle progression [12], we also investigated the expression levels of c-Myc by immunoblot assay in luteolin treated HeLa cells. Consistent with above findings luteolin dose-dependently reduced c-Myc protein level (Fig. 1A and B). Altogether, these results showed that luteolin apparently suppressed STAT3 transcriptional activity through reducing phosphorylated STAT3 level.

### Hsp90 Provides a Resistance to Luteolin-induced Interacting Proteins Degradation

**Figure 2:**
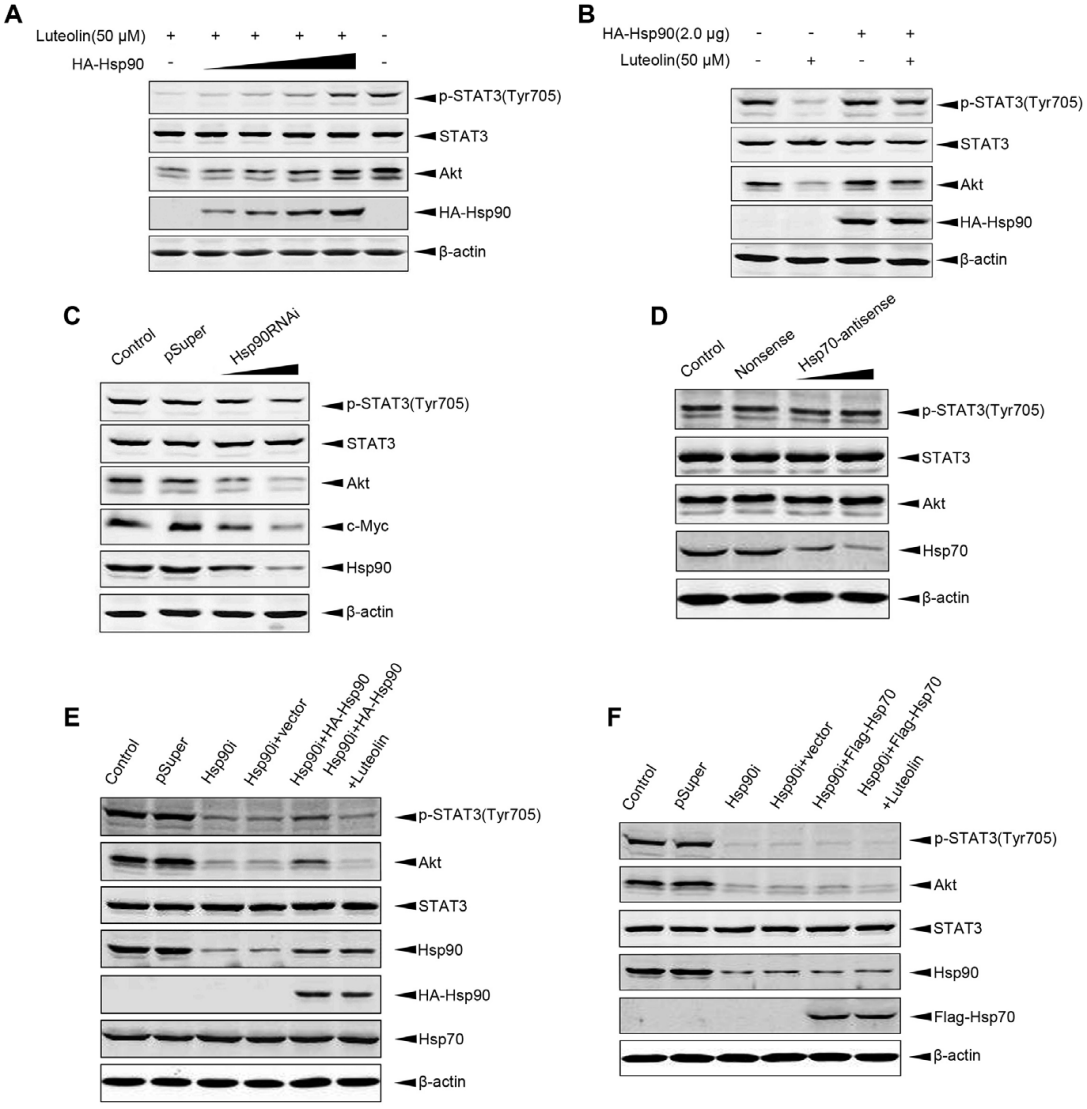
Hsp90 reversed luteolin-induced phosphor-STAT3 and Akt down-regulation. A, HeLa cells were transfected with 0.5, 1, 1.5, 2 µg HA-Hsp90 plasmids and incubated with (+) or without (−) 50 µM luteolin for 24 h before harvesting. Western blotting with specific antibodies was performed for p-STAT3, STAT3, and Akt respectively. B, HeLa cells were transfected with HA-Hsp90 (2 µg) or empty vectors for 24 h, and then cells were treated with 50 µM luteolin for 24 h. The endogenous Akt, STAT3 and Tyr^705^-phosphorylated-STAT3 were measured by immunoblot analysis respectively. C, HeLa cells were transfected with pSuper-Hsp90i or pSuper vector, and then were subjected to immuoblot analysis for measuring indicated proteins. D. As a control, HeLa cells were transfected with Hsp70 nonsense or antisense oligonucleotides, and then were analyzed by western blotting using anti-p-STAT3, anti-STAT3, anti-Akt antibodies. E, HeLa cells were first transfected with pSuper-Hsp90i or pSuper, and 24 h later, cells were transfected with HA-Hsp90 or vector. And then cells were treated with luteolin for 24 h. Lysates were subjected to immunoblotting for testing p-STAT3, STAT3, Akt, Hsp90. F, HeLa cells were first transfected with pSuper-Hsp90i and then transfected with HA-Hsp70. After 24 h cells were treated with luteolin for 24 h, and subjected to immunoblotting for detecting indicated protein levels.

Hsp90 may function as a stabilizer of phosphorylated STAT3 by directly interacting with it [14]. Since luteolin decreased phosphorylated STAT3, we then evaluated whether luteolin could act on hsp90. We transiently transfected HeLa cells with increasing concentrations of hemagglutinin (HA)-tagged Hsp90 or empty vector. Twenty four hours after transfection, cells were treated with 50 µM luteolin or ethanol for another 24 h. The results showed that overexpression of HA-Hsp90 dose-dependently inhibited the degradation of Tyr705-phosphorylated STAT3 and Akt induced by luteolin (Fig. 2A and B). Akt was a known client proteins of Hsp90. To further confirm the effects of luteolin on Hsp90, we then transfected plasmids of pSuper-Hsp90i into HeLa cells to knock down endogenous Hsp90. As shown in fig. 2C, endogenous Hsp90 expression was efficiently silenced. Hsp90 RNAi obviously strengthened the effect of luteolin on down-regulation of Tyr^705^-phosphorylated STAT3 and Akt(another Hsp90 client protein). Moreover, c-Myc was also downregulated, as the consequence of Tyr^705^-phosphorylated STAT3 reduction by Hsp90 RNAi (Fig. 2C). However, transfection of Hsp70 nonsense or antisense oligonucleotides did not affect these protein levels (Fig. 2D). We restored a portion of Hsp90 after Hsp90 RNAi and then treated cells with luteolin. As shown in Fig. 2E, when the cells were forced to express HA-Hsp90 followed by silence of endogenous Hsp90, transfection of Hsp90 resulted in the recovery of p-STAT3 (Tyr705) and Akt. Nevertheless, these restored client proteins of Hsp90 reduced after the cells being treated with luteolin (Fig. 2F). These results strongly suggested that luteolin decreased Tyr^705^-phosphorylated STAT3 by disrupting the function and inhibiting the activity of Hsp90.

### Luteolin Induces Hsp90 Interacting Proteins Degradation through Ubiquitin-proteasome-dependent Pathway

**Figure 3:**
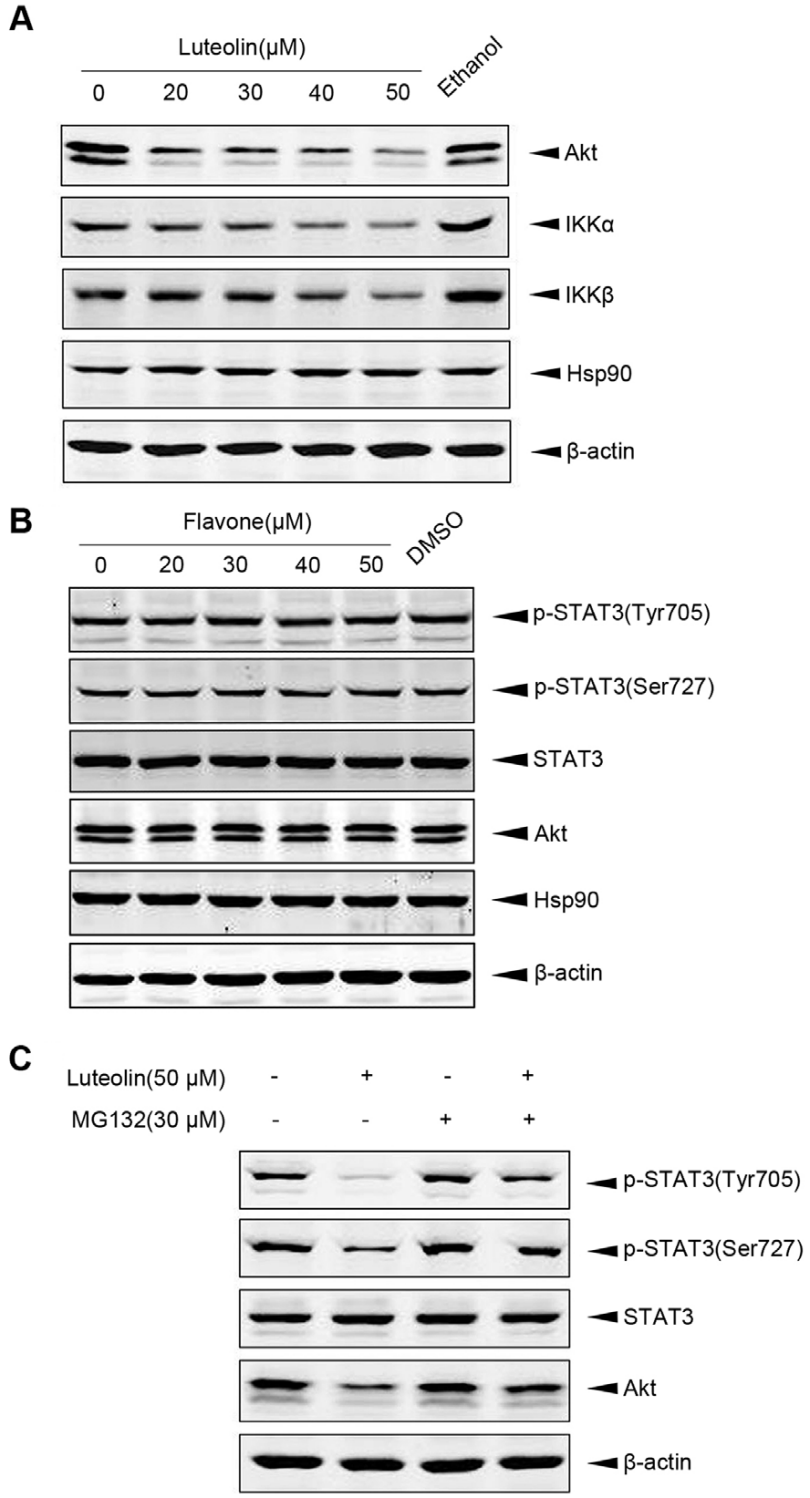
Luteolin down-regulated interacting proteins of Hsp90 and induced degradation of prospho-STAT3 and Akt. A, HeLa cells were treated with 0, 20, 30, 40, 50 µM luteolin for 24 h and then were lysed. Protein levels of Akt, IKKα, IKKβ and Hsp90 in cell lysates were detected by using indicated antibody respectively. B, As a control, indicated dose of flavone was used to treat HeLa cells for 24 h. The levels of phosphor-STAT3 (tyr705), phosphor-STAT3 (ser727), STAT3, Akt and Hsp90 were detected by Western blotting. C, HeLa cells were treated with luteolin (50 µM) for 24 h in the presence of proteasome inhibitor MG-132 (30 µM), protein levels of phosphor-STAT3 (tyr705), phosphor-STAT3 (ser727), STAT3 and Akt were measured by Western blot analysis.

As STAT3 has been reported to be a client protein of Hsp90 [21] and above data demonstrated that luteolin decreased Tyr^705^-phosphorylated STAT3, we observed whether other Hsp90 client proteins could also be affected by luteolin. HeLa were treated with indicated doses of luteolin for 24 h and then were subjected to immunoblot analysis. As expected, luteolin dose-dependently decreased the amount of endogenous Akt, IKKα and IKKβ, but did not reduce Hsp90 level (Fig. 3A). These data suggested that luteolin might inhibit molecular chaperone activity of Hsp90 and then decreased its client proteins. In comparison with luteolin, flavone, the nonhydroxylated core structure of the flavones, showed no effect on these Hsp90 client proteins, which suggested the specific effect of luteolin on Hsp90 (Fig. 3B). It is believed that luteolin promoted degradation of Tyr^705^-phosphorylated STAT3 in the ubiquitin-proteasome-dependent manner [9]. Our results showed that there was a potent increase of the Tyr^705^-phosphorylated STAT3, Ser^727^-phosphorylated STAT3 and Akt in cells co-treated with luteolin and MG-132 compared with those cells treated with luteolin only (Fig. 3C). This result suggested that luteolin promoted the proteasome-dependent degradation of Hsp90 client proteins.

### Luteolin Prevents the Association between Hsp90 and STAT3

**Figure 4:**
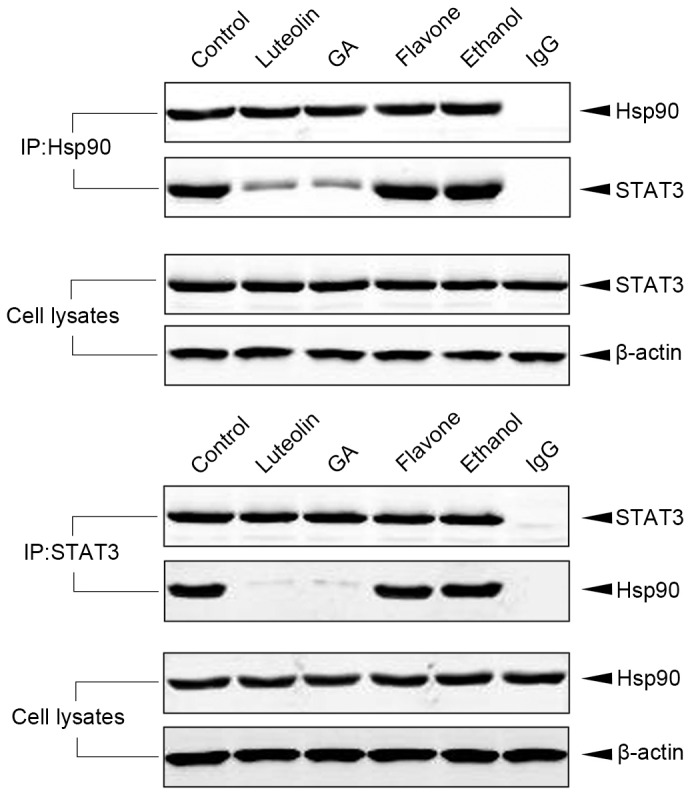
Luteolin inhibited the association between Hsp90 and STAT3. HeLa cells were treated with luteolin, GA, flavones, ethanol or left untreated respectively. Cell laysates were immunoprecipitated with anti-Hsp90 antibody or control rabbit IgG, and the immunopellets were detected by immunoblot analysis with anti-STAT3 antibody. Cell laysates were then immunoprecipitated with anti-STAT3 antibody or control rabbit IgG, and the immunopellets were detected by immunoblot analysis with the anti-Hsp90 antibody.

As a molecular chaperone, Hsp90 stabilized its client proteins by forming complexes with them. The inhibitors of Hsp90, such as GA, could lead to dissociation of Hsp90 from its client proteins and induce these proteins degradation. We therefore observed that whether luteolin could affect the complex of Hsp90 and STAT3. HeLa cells were treated with luteolin, GA, flavone or ethanol respectively, and then subjected to co-immunoprecipitation and immunoblot analysis. The results demonstrated that in luteolin and GA treated cells, the complex of Hsp90 and STAT3 significantly decreased. These data indicated that luteolin inhibited the capability of Hsp90 for associating with its client proteins (Fig. 4).

### Luteolin Interacts with Hsp90

**Figure 5:**
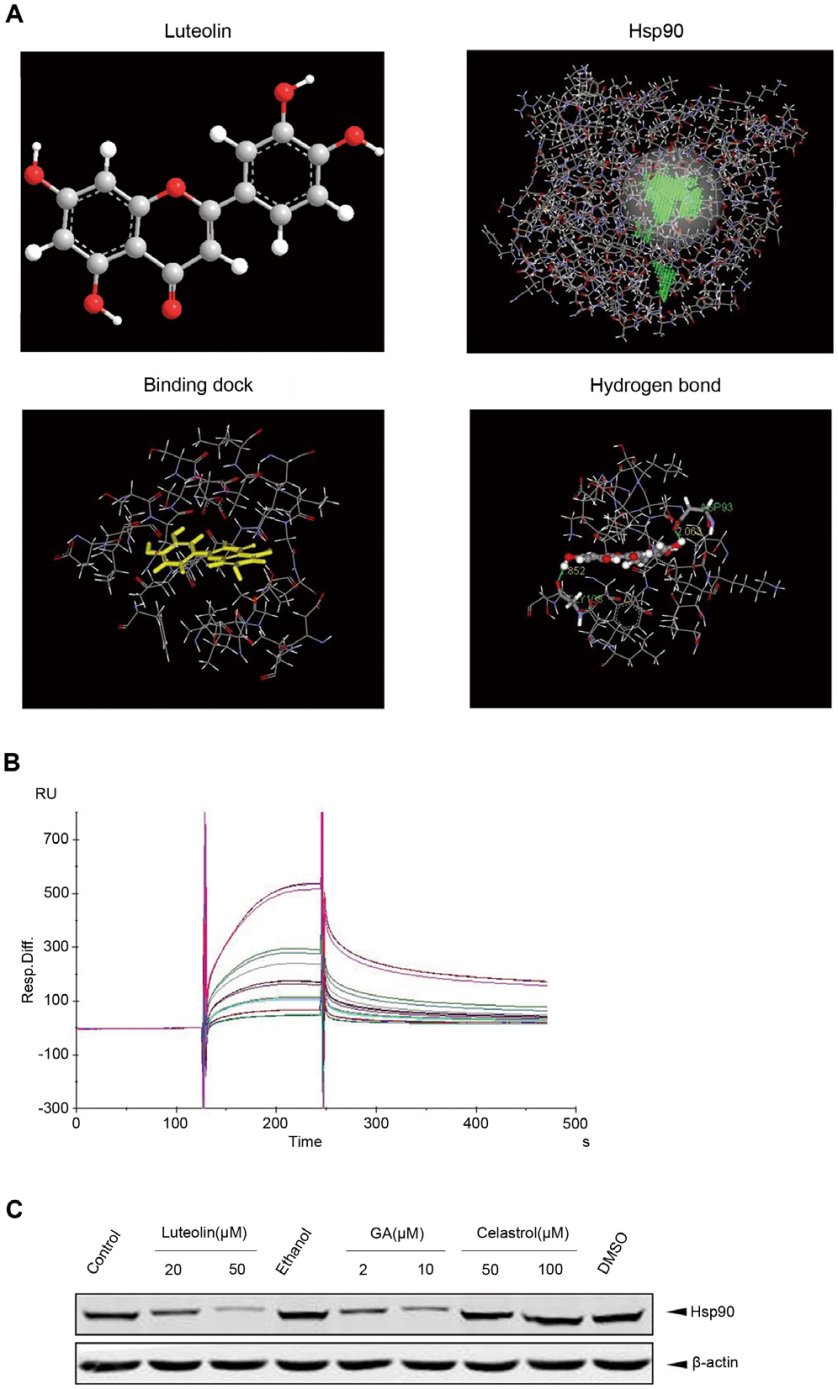
Luteolin associated with Hsp90. A. Upper left picture showed the chemical structure of luteolin. Upper right figure displayed the structure of Hsp90 with its two ATPase sites (in green). The lower left picture showed the molecular modeling of binding sites in Hsp90 for luteolin performed by using Chemistry at HARvard Macromolecular Mechanics (CHARMm)-Version 33.1. The lower right figure displayed hydrogen bonds between Hsp90 and luteolin (in green). B. SPR analysis showed the interaction between different concentration of luteolin and Hsp90. C. HeLa cell were incubated with luteolin, geldanamycin, celastrol, ethanol, DMSO or left untreated. Pre-equilibrated γ-phosphatelinked ATP-Sepharose was used to pull down endogenous Hsp90. ATP-Sepharose beads bound Hsp90 was detected by immunoblotting.

The inhibitors of Hsp90, such as GA, inhibited Hsp90 activity by binding to Hsp90. We next evaluated whether luteolin interacted with Hsp90. Utilizing the crystal structure of Hsp90, we analyzed the model of association between luteolin and Hsp90. In accordance with previous reports, our molecular modeling showed that there were two ATP binding domain in the N-terminal and C-terminal region of Hsp90 respectively. The chemical structure of luteolin was displayed in Fig. 5A (upper left). As shown in Fig. 5A, upper right, the two green circles displayed the two areas with different ATPase activities, and the N-terminal ATPase site possessed a higher ATP/ADP binding activity. The binding possibility between Hsp90 and luteolin was evaluated by CHARMm–Discovery Studio 2.1(DS 2.1). According to the analysis to pH condition and changes of the molecular, we got the most steady state of binding between luteolin and Hsp90. Based on the molecular modeling it is demonstrated that luteolin could bind to N-terminal ATPase site of Hsp90 (Fig. 5A, lower left). The amino acid residues involved in the binding of luteolin to Hsp90 and the hydrogen bond between Hsp90 and luteolin were predicted in Fig. 5A (upper right). The hydrogen bonds between hydrogen atoms were strong. The fairly strong hydrogen bonds were formed by the interaction of the oxygen atoms in luteolin molecules with Asp93 and Gly103 of Hsp90 respectively. The two types of strong hydrogen bonds were probably the important driving force, which induced distortion of the structure of the steering group. The interaction between luteolin and Hsp90 was not exclusive because there were several hydrogen atoms as well as residues around luteolin (Fig. 5A, lower right). These polar residues might play an important role in stabilizing drag via H-bonds and electrostatic interactions. The results from the molecular modeling revealed that the hydrogen bonds were main binding force between the luteolin and Hsp90. The hydrogen bonds or electrostatic interaction acted as an anchor which helped luteolin to attain the 3D space position inATP-binding pocket of Hsp90. To inspect if luteolin really could interact with Hsp90, SPR technology-based Biacore X100 biosensor was used (GE Healthcare, Piscataway, NJ). The SPR analysis indicated that luteolin really could bind to Hsp90 (Fig. 5B) To confirm that lutoelin could occupy ATP-binding pocket of Hsp90, we employed the ATP-sepharose pull-down assay. As shown in Fig. 5C, ATP-sepharose beads successfully pulled down Hsp90 in solution. Like GA, the ATPase activity inhibitor of Hsp90, luteolin decreased Hsp90 pulled down by ATP-sepharose beads suggesting that luteolin was able to block Hsp90-ATP binding. In contrast, celastrol, an Hsp90 inhibitor without ATPase inhibition, did not block ATP binding with Hsp90. Altogether, these data indicated that luteolin could bind to Hsp90 to restrain the binding of Hsp90 with ATP competitively, thereby inhibiting Hsp90 chaperone activity.

### Luteolin Induces Apoptosis of Cancer Cells

**Figure 6:**
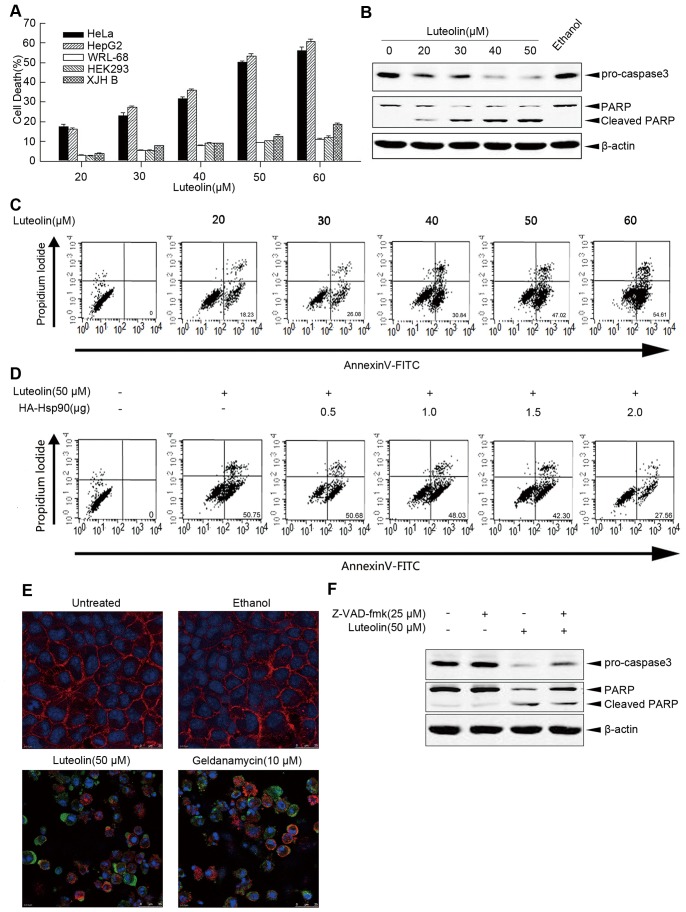
Luteolin induced carcinoma cells apoptosis. A, Carcinoma cells (HeLa, HepG2), and normal cells (WRL-68, HEK293, XJH B) were treated with 20, 30, 40, 50, 60 µM luteolin for 24 h and followed by CytoTox-Glo™ cytotoxicity assays. The data above are plotted as “dead cell” rate versus the concentration of Luteolin. B. HeLa cells were treated with indicated concentration of luteolin for 24 h and subjected to immunoblot analysis for pro-caspase3 and PARP. C. HeLa cells were treated with indicated concentration of luteolin for 24 h and were harvested, and then stained with propidium iodidle and AnnexinV-FITC for detecting the apoptosis by flow cytometry. D. HeLa cells were transfected with indicated concentration of HA-Hsp90 and 24 h after transfection cells were treated with 50 µM luteolin for 24 h and were harvested, and then stained with propidium iodidle and AnnexinV-FITC for detecting the apoptosis by flow cytometry. E. HeLa cells were treated with 50 µM luteolin and 10 µM GA, for 24 h, and then cells were incubated with primary antibodies against PARP. HeLa cells were immunostained with anti-mouse alexa fluor 488 secondary antibody and then stained with DAPI and Alexa Fluor 555 phalloidin derivatives for labeling F-actin. The specimens were visualized by confocal laser scanning microscopy. Blue depicts the nucleus, red depicts localization of F-actin and green depicts localization of cleaved-PARP. F. HeLa cells were pretreated with 25 µM z-VAD-fmk for 1 h and then treated with 50 µM luteolin for 24 h followed by immunoblotting with pro-caspase3 and cleaved PARP antibodies.

GA and other Hsp90 inhibitors have been considered to be used as anticancer drugs. We compared the effect of luteolin on human normal cells and cancer cells by using CytoTox-Glo™ Cytotoxicity assay. The results demonstrated that luteolin showed a dose-dependent cytotoxicity to cancer cells including HeLa and HepG2, but showed a very slight cytotoxicity to normal cells, such as WRL-68, HEK293 and XJH cells [31] (Fig. 6A), which suggested that luteolin possessed potential ability to induce cancer cell death.

Because apoptosis is usually associated with activation of caspases [32], western blot analysis was used to detect the activation of pro-caspase-3 and the cleavage of the caspase-3 substrate PARP in luteolin-treated HeLa cells. Luteolin treatment caused a conspicuous activation of precursor caspase-3 and an increase of cleavage of PARP (Fig. 6B). Luteolin treated-HeLa cells were also stained with annexin V/PI followed by flow cytometry assay. HeLa cells showed a clear apoptosis within 24 h after luteolin treatment (Fig. 6C), and overexpression of Hsp90 prevented luteolin-induced HeLa cell apoptosis (Fig. 6D). Consistent with this finding, the caspase inhibitor carbobenzoxy-valyl-alanyl-aspartyl-[O-methyl]-fluoromethylketone (Z-VAD-FMK) significantly inhibited the luteolin-induced apoptosis (Fig. 6F). These results suggested that luteolin-induced cytotoxicity in carcinoma cells is associated with apoptosis [9].

To compare the anticancer effect of luteolin with GA, we treated HeLa cells with 50 µM luteolin or 10 µM GA and observed morphological changes in luteolin and GA treated cells by DAPI and Alexa Fluor^®^ 555 phalloidin staining followed by confocal IF analysis. As shown in Fig. 6E, both luteolin and GA treated cells showed some features related with cell death, such as cell shrinkage, cell membrane blebbing, and nuclear condensation, while no significant change was found in ethanol treated HeLa cells.

## Discussion

Previous studies have shown that overexpression of Hsp90 in cancer cells was related to the drug resistance and poor response to chemotherapy agents [21]. Many client proteins of Hsp90 contribute to the hallmarks of cancer including self-sufficiency ingrowth signals, evasion of apoptosis and limitless replicative potential [33]. Inhibition of Hsp90 mediated conformational maturation/refolding reaction will cause the degradation of Hsp90 substrates [19]. Hsp90 plays a vital role in human tumors for chaperoning the oncoproteins to maintain their oncogenic function. It is reported that Hsp90 showes 20 to 200 times higher binding affinity for inhibitors in tumor cells than normal cells because of its high ATPase activity in tumor cells [34]. Due to its unique role in stabilizing oncogenic proteins, Hsp90 is considered to be an important target in cancer therapy [18]. The ATPase activity center of Hsp90 located in the N-terminal domain can be regulated. The identification of co-chaperones that facilitate Hsp90 function were landmarks towards understanding conformational changes in Hsp90 brought about by ATP, co-chaperones and interacting proteins [35]. The co-crystallization reveals that GA binds to a pronounced pocket of Hsp90, which is the ATP-binding site in the N-terminal domain of Hsp90 [36], [37].

A new Hsp90_ inhibitors, CLC107 (the derivatives of YC-1), was investigated for its potent anticancer activity and its ability to deplet the HER2/neu expression through inhibiting Hsp90. In this study, luteolin showed high affinity to Hsp90 by one H bond with Asp93 (distance: 2.10 Å) and one with Asn51 (distance: 2.42 Å) [38]. In our study, molecular modeling analysis indicated that luteolin could bind to the N-terminal ATP/ADP-binding domain of Hsp90. As expected, SPR analysis displayed the stability of interaction between luteolin and Hsp90. Further observation indicated that luteolin significantly inhibited ATP-Hsp90 binding, which strongly suggested that luteolin inhibited ATPase activity of Hsp90. If Hsp90 was a critical target of luteolin, overexpression of Hsp90 should attenuate luteolin-induced protein degradation. As we assumed, overexpression of HA-Hsp90 dose-dependently inhibited the degradation of Tyr^705^-phosphorylated STAT3 and Akt induced by luteolin.

Early study has reported that luteolin promoted the ubiquitin-dependent degradation in Tyr^705^-phosphorylated STAT3 [9], thus it down-regulated the survivin and up-regulated the Fas/CD95. However, this study did not involve the effect of luteolin on Hsp90. In our further investigations,we found that luteolin decreasedthe level of Tyr^705^-phosphorylated STAT3, as well as some other Hsp90 client proteins including Akt and IKK. Furthermore, luteolin promoted the proteasomal degradation of client proteins of Hsp90. Our Molecular modeling and SPR analysis indicated that luteolin could bind to the N-terminal ATP/ADP-binding domain of Hsp90. Further observation indicated that luteolin significantly inhibited ATP-Hsp90 binding strongly suggesting that luteolin inhibited ATPase activity of Hsp90.

GA has been thought to possess significant antitumor activity in human tumor cells, but due to its intolerable toxicity, GA has not been used in clinic. Though 17-AAG shows reduced hepatotoxicity, its antitumor activity is relatively weak [39]. It has been reported that some flavonoids possess anticancer activities at almost nontoxic concentrations [40]. The mechanisms of their anticancer effects have been detected, including carcinogen inactivation, antiproliferation, cell cycle arrest, induction of apoptosis and differentiation, inhibition of angiogenesis, antioxidation, reversal of multidrug resistance and a combination of these mechanisms [5]. These encouraging results from previous investigations stimulated the clinical trials of flavonoids in human. Phase I clinical study with quercetin (3,3′,4′,5,7-pentahydroxy-flavone) for the treatment of cancer has been performed [41]. However, because of its high concentration (1400 mg/m^2^) needed, quercetin was not suitable for intravenously administration in clinical use [5], [41]. It has been reported that luteolin exerted the anticancer effects by suppressing cancer cell growth and migration [42], [43], [44]. In the present study, we found that luteolin induced HeLa cell apoptosis through promoting degradation of phosphorylated STAT3. Block of STAT3 Tyr^705^-phosphorylation might disrupt STAT3 dimer formation and transcriptional activity, therefore induce apoptosis of STAT3-positive carcinoma cells. Cancer cells exhibit a higher dependence on Hsp90 than normal cells, therefore, block of Hsp90 activity will critically interfere cancer cell but not normal cells. It is not surprised that luteolin induced apoptosis of cancer cell but just showed slight cytotoxicity to normal cells.

In conclusion, our findings highlighted a novel mechanism for luteolin to induced apoptosis of carcinoma cells. Luteolin bound to Hsp90 and induced its client proteins dissociate from Hsp90 and promoted degradation of some key antiapoptotic proteins such as activated STAT3 and Akt [45], [46], [47], and then induced apoptosis of carcinoma cells. Our results provide an important insight for understanding the molecular mechanism of the anticancer effect of luteolin. Because of the importance of Hsp90 in oncogenesis, these findings will be helpful to recognize that luteolin may be a potent inhibitor for Hsp90 in antitumor strategies.
